# Geographic range estimates and environmental requirements for the harpy eagle derived from spatial models of current and past distribution

**DOI:** 10.1002/ece3.7068

**Published:** 2020-12-15

**Authors:** Luke J. Sutton, David L. Anderson, Miguel Franco, Christopher J. W. McClure, Everton B. P. Miranda, F. Hernán Vargas, José de J. Vargas González, Robert Puschendorf

**Affiliations:** ^1^ School of Biological and Marine Sciences University of Plymouth Plymouth UK; ^2^ The Peregrine Fund Boise ID USA; ^3^ University of KwaZulu‐Natal Pietermaritzburg South Africa

**Keywords:** geographic range size, *Harpia harpyja*, harpy eagle, Neotropical raptors, point process models, species distribution models

## Abstract

Understanding species–environment relationships is key to defining the spatial structure of species distributions and develop effective conservation plans. However, for many species, this baseline information does not exist. With reliable presence data, spatial models that predict geographic ranges and identify environmental processes regulating distribution are a cost‐effective and rapid method to achieve this. Yet these spatial models are lacking for many rare and threatened species, particularly in tropical regions. The harpy eagle (*Harpia harpyja*) is a Neotropical forest raptor of conservation concern with a continental distribution across lowland tropical forests in Central and South America. Currently, the harpy eagle faces threats from habitat loss and persecution and is categorized as Near‐Threatened by the International Union for the Conservation of Nature (IUCN). Within a point process modeling (PPM) framework, we use presence‐only occurrences with climatic and topographical predictors to estimate current and past distributions and define environmental requirements using Ecological Niche Factor Analysis. The current PPM prediction had high calibration accuracy (Continuous Boyce Index = 0.838) and was robust to null expectations (pROC ratio = 1.407). Three predictors contributed 96% to the PPM prediction, with Climatic Moisture Index the most important (72.1%), followed by minimum temperature of the warmest month (15.6%) and Terrain Roughness Index (8.3%). Assessing distribution in environmental space confirmed the same predictors explaining distribution, along with precipitation in the wettest month. Our reclassified binary model estimated a current range size 11% smaller than the current IUCN range polygon. Paleoclimatic projections combined with the current model predicted stable climatic refugia in the central Amazon, Guyana, eastern Colombia, and Panama. We propose a data‐driven geographic range to complement the current IUCN range estimate and that despite its continental distribution, this tropical forest raptor is highly specialized to specific environmental requirements.

## Graphical abstract

Understanding species–environment relationships is key to defining the spatial structure of species distributions and develop effective conservation plans. The harpy eagle (*Harpia harpyja*) is a large raptor with a continental distribution across the Neotropics, categorized as Near‐Threatened by the International Union for the Conservation of Nature (IUCN). We propose a data‐driven geographical range to complement the current IUCN range estimate and that despite its continental distribution, this tropical forest raptor is highly specialized to specific environmental requirements.

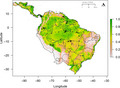

## 1 INTRODUCTION

Defining species distributions in geographic and environmental space is a fundamental component of conservation management (Peterson et al., 2011). Yet this information is lacking for many rare and threatened taxa in a rapidly changing environment (Lawler et al., 2011; Miller, 2010). Assessing geographic distribution and environmental requirements of rare, poorly studied and cryptic species can be problematic due to scarce occurrence data, resulting in limited information for conservation managers to act upon (Pearce & Boyce, 2006). For these underdocumented species, this baseline spatial information is either inadequate, or nonexistent, especially in highly biodiverse tropical regions, often where organismal biology is also poorly known (Buechley et al., 2019; Rodríguez et al., 2007; Tobias et al., 2013; Wilson et al., 2016). In response to this knowledge gap, spatial modeling techniques have been developed to help direct conservation actions and implement research programs.

Species distribution models (SDMs) can overcome deficiencies in information regarding distribution by correlating the underlying environmental data at known occurrences to predict the areas of highest environmental suitability (Elith & Leathwick, 2009; Scott et al., 2002). On the other hand, ordination approaches define the underlying environmental factors that explain the most suitable environmental conditions for where a given species is found. Combining both SDMs and ordination is an effective method to define the distributional and ecological constraints of a given species (Chase & Leibold, 2003; Peterson et al., 2011; Soberón & Nakamura, 2009). These methods are particularly useful when using species occurrences generated from biodiversity databases when modeling distributions for species in remote, difficult to survey regions (Peterson, 2001; Rhoden et al., 2017; Sutton & Puschendorf, 2020).

The Neotropics are well‐known for high avian biodiversity. Yet many birds, including raptors, face multiple threats across the area, largely driven by human activities such as habitat loss, agricultural development, and resource overexploitation (Buechley et al., 2019; McClure et al., 2018; Sarasola et al., 2018; Tobias et al., 2013). Due to the difficulties of sampling across the extensive and complex terrain of the Neotropics, applying SDMs using open‐access distribution data can generate baseline information on species distributions in a rapid and cost‐effective manner (Cayuela et al., 2009; La Sorte & Somveille, 2020). The harpy eagle (*Harpia harpyja*) is a large Neotropical raptor, with a broad yet shrinking range across Central and South America from southern Mexico to northern Argentina (Ferguson‐Lees & Christie, 2005; Vargas González et al., 2006). Harpy eagles generally occur at low population densities in lowland tropical forest (Vargas González & Vargas, 2011) but are nearly extinct in Brazil's Atlantic forest (Meller & Guadagnin, 2016; Srbek‐Araujo & Chiarello, 2006) and in forest enclaves such as riparian forests in open savannahs (Silva et al., 2013).

With generally low population densities and a 3‐year long breeding cycle, the harpy eagle is considered a species of conservation concern due to continued habitat loss and persecution (Miranda et al., 2019; Vargas González et al., 2006). Currently categorized as “Near‐Threatened” by the International Union for the Conservation of Nature (IUCN; Birdlife International, 2017), local extirpations have occurred in most of Central America, and the population status of the species across its continental range is largely unknown (Vargas González et al., 2006). The current IUCN geographic range for the harpy eagle estimates an extent of occurrence (EOO) of 17.6 million km^2^ and an unknown Area of Occupancy (AOO, Birdlife International, 2017). EOO measures the area within a minimum convex polygon (MCP) from all known species occurrences, while AOO is a subset of the EOO where the species actually occurs in occupied grid cells of 2 × 2 km, excluding vagrancy (Brooks et al., 2019; Gaston & Fuller, 2009). Both measures are based solely on spatial locations and not on underlying environmental information.

One of the main criticisms of using EOO is that it often includes unsuitable areas, overestimating the true range, which is more likely to show a discontinuous pattern of distribution (Breiner et al., 2017; Jetz et al., 2007; Peterson et al., 2016; Ramesh et al., 2017). SDMs are useful as an alternative measure to complement IUCN estimates, intermediate between EOO and AOO, especially for rare and undersampled species (Breiner et al., 2017). SDMs should not be viewed as surrogates for IUCN criteria but can provide a basis for estimating AOO (Breiner et al., 2017; Gaston & Fuller, 2009; IUCN, 2019), especially in the case for the harpy eagle where this figure is unknown. Using the underlying environmental signature of the species as a guide for model interpolation may produce a more realistic data‐driven estimate of distribution area (Peterson et al., 2016). Global range size is a key parameter for assessing threat status and extinction risk; thus, overestimating this figure could lead to increasingly threatened species being missed (Ramesh et al., 2017). Predicting areas with the highest environmental suitability can thus focus research effort and update threatened species' conservation status (Bierregaard, 1998).

Miranda et al. (2019) produced the first SDM for the harpy eagle, identifying its close relationship to lowland tropical forest. We build on the strengths of this initial SDM, first by incorporating extra presence‐only occurrences with the Miranda *et al*. location data, and second using an expanded set of environmental predictors. Additionally, we project current predictions into two paleoclimatic scenarios and predict how past distributions may influence present and future distribution. Long‐term ecological perspectives from paleoclimate models are important for comparing current distribution to past fluctuations (Fuller et al., 2011; Nogués‐Bravo, 2009). Further, having a long‐term perspective of past distributions is critical to interpreting current distribution and can point toward potential refugia expected from future changes in range size (Fuller et al., 2011; Keppel et al., 2012). Understanding the species–environment relationships regulating current and historical harpy eagle distribution can therefore help direct conservation management by identifying the spatial extent for the species.

Here, predictive spatial models are developed for the harpy eagle in geographic space using a point process modeling (PPM) framework. Recently, PPMs have been shown to be most effective for modeling distributions using presence‐only occurrences (Renner et al., 2015; Warton & Shepherd, 2010). PPMs model the intensity of occurrence points across a given area, thus under low spatial dependence of occurrences the resulting outputs can be interpreted as either the relative (Renner et al., 2015), or potential abundance of focal species (Phillips et al., 2017). An ecological profile is then developed using ordination with an Ecological Niche Factor Analysis (ENFA) to best explain the environmental requirements of the harpy eagle, compared to the background environmental conditions available. Specifically, we aim to (a) re‐evaluate current harpy eagle distribution and establish its ecological niche as a function of climatic and topographical predictors, (b) revise the estimated current coarse‐scale IUCN distributional area and provide complementary range maps, and (c) predict past distributions from two paleoclimatic time periods and combine with the current model to identify stable refugia.

## 2 MATERIALS AND METHODS

### 2.1 Harpy eagle occurrence data

Harpy eagle occurrences were sourced from the Global Raptor Impact Network (GRIN, The Peregrine Fund, 2018) a data information system for all raptor species. For the harpy eagle, GRIN consists of occurrence data from the Global Biodiversity Information Facility (GBIF, 2019), which are mostly eBird records (89.88%, Sullivan et al., 2009), combined with two additional datasets of nests and observations (Miranda et al., 2019; Vargas González & Vargas, 2011). Occurrence data were cleaned by removing duplicate records, those with no geo‐referenced location and for spatial auto‐correlation (see Appendix 1 in Supporting Information). To account for sampling bias in occurrences, a 4 km spatial filter from each occurrence point was used to minimize the effects of survey bias, using the “thin” function in the R package spThin (Aiello‐Lammens et al., 2015). The 4 km thinning distance was selected as a proxy of mean internest distances based on breeding pairs in the Darien region of Panama (Vargas González & Vargas, 2011). We used 4 km as a minimum distance knowing that internest distances recorded across the harpy eagle range can vary (Muñiz‐López, 2008; Piana, 2007). After data cleaning, a total of 1,179 geo‐referenced records were compiled for inclusion in model calibration, generally within the current range defined by the IUCN (Figure S1, see Appendix 3 in Supporting Information; Birdlife International, 2017). Applying the 4 km spatial filter resulted in 742 occurrence records for use in the calibration models. The resulting occurrence points are thus best reported as locations in continuous space, providing the primary motivation for using the PPM regression framework for subsequent spatial analysis (Renner et al., 2015).

### 2.2 Environmental predictors

Thirty‐seven bioclimatic and topographical predictors were obtained from the WorldClim (v1.4, Hijmans et al., 2005) and ENVIREM (Title & Bemmels, 2018) databases. WorldClim variables (*n = *19) are generated through interpolation of average monthly weather station climate data from 1960 to 1990. The ENVIREM dataset includes 16 climatic and two topographic variables to complement the WorldClim dataset providing a wider range of potential variables from which to select model predictors. Raster layers were cropped and masked to a delimited polygon consisting of all known range countries (including the states of Formosa, Jujuy, Misiones and Salta in northern Argentina, and the states of Chiapas, Oaxaca, and Tabasco in southern Mexico), to extend into potential areas of marginal habitat on the distribution edges. Reducing the accessible area to the known range improves model predictive power by reducing the background area used for testing points used in model evaluation (Barve et al., 2011; Radosavljevic & Anderson, 2014).

For past predictions, three general circulation models (GCMs, Table 1) were used from the Coupled Model Intercomparison Project Phase 5 (CMIP5) and Paleoclimate Modeling Intercomparison Project Phase 3 (PMIP3) databases for two paleoclimate scenarios in the Mid‐Holocene (~6,000 cal yr BP) and Last Glacial Maximum (~22,000 cal yr BP). Three GCMs were used to account for variation and uncertainty in model predictions (Nogués‐Bravo, 2009), and a summed prediction calculated from all models for both paleoclimate scenarios. Each summed paleo‐distribution was then stacked with the current distribution and overlaid to provide a summed estimate of environmental stability (Peterson et al., 2017), using the “stability” function in the R package “sdStaf” (Atauchi, 2018). Summed stability can predict areas of stable refugia, where a species is predicted to be present irrespective of time period (Carnaval et al., 2009). Geographic niche overlap from the individual GCMs was tested using Schoener's *D* (Schoener, 1968; Warren et al., 2008), which ranges between 0 (no overlap) and 1 (identical overlap). Paleoclimate raster data were downloaded from the WorldClim (v1.4, Hijmans et al., 2005) and ENVIREM (Title & Bemmels, 2018) databases and masked to the current range extent to predict areas of past climatic suitability compared to the current range.

**Table 1:** General Circulation Models (GCMs) from the Coupled Model Intercomparison Project Phase 5 (CMIP5) and Paleoclimate Modeling Intercomparison Project Phase 3 (PMIP3) databases used to predict past distributions for the harpy eagle to two paleoclimate scenarios in the Mid‐Holocene (~6,000 cal yr BP) and Last Glacial Maximum (~22,000 cal yr BP)

GCM	Acronym	Citation
Community Climate System Model, v4	CCSM4	Gent et al. (2011)
Model for Interdisciplinary Research on Climate		
– Earth System Model	MIROC‐ESM	Watanabe et al. (2011)
Max Planck Institute for Meteorology		
– Earth System Model ‐ Paleo	MPI‐ESM‐P	Giorgetta et al. (2013)

Multicollinearity between environmental predictor variables can bias models by over‐representing the biological relevance of correlated variables (Franklin, 2009; Phillips et al., 2006). Before model construction, environmental cells containing occurrence records from all 37 variables were tested for multicollinearity using Variance Inflation Factor (VIF) analysis (Guisan et al., 2006; Hair et al., 2006) with the “corSelect” function in the R package fuzzySim (Barbosa, 2015, 2018). A stepwise elimination of highly correlated variables was used retaining predictors with a VIF threshold < 10 considered as suitable for multi‐variable correlation (Dormann et al., 2013). The remaining variables were then checked for collinearity using Spearman's correlation coefficient with only variables *r_s_ *≤ |0.7| retained for consideration as predictors. We used solely climatic and topographical predictors as to our knowledge there are no reliable estimates of landcover extent or anthropogenic impact extending back to the two paleoclimate scenarios used here.

After removing highly correlated variables, eight climatic variables (isothermality; maximum temperature warmest month; precipitation wettest month; precipitation warmest quarter; Climatic Moisture Index (CMI); minimum temperature warmest month, potential evapotranspiration (PET) driest quarter; PET wettest quarter) and one topographic variable, Terrain Roughness Index (TRI), were included as predictors at a spatial resolution of 2.5 arc‐minutes (~4.5 km resolution). Final predictor selection was based on representing monthly and seasonal climatic trends, extremes and limiting environmental factors strongly related theoretically and empirically to species distributions (Stockwell, 2006; Bradie & Leung, 2017; Guevara et al., 2018; see Appendix 1 in Supporting Information). For example, in tropical forests, rainfall regime and seasonality are predicted to have a strong effect on avian survival, food availability, and reproductive effort (Stotz et al., 1996; Williams & Middleton, 2008). Therefore, predictors were selected based on seasonal and monthly precipitation interacting with temperature, as potential limiting factors on harpy eagle distribution (Busch et al., 2011; Williams & Middleton, 2008).

### 2.3 Species distribution models

SDMs were fitted using a point process modeling (PPM) framework as a form of infinitely weighted logistic regression via penalized maximum likelihood (Fithian & Hastie, 2013), treating occurrences as points rather than grid cells in the R package maxnet (Phillips et al., 2017) and maximum entropy software, MAXENT (v3.4.1). Recent theoretical work has demonstrated the equivalence of MAXENT to an inhomogeneous Poisson process (IPP; Fithian & Hastie, 2013; Renner et al., 2015; Renner & Warton, 2013), which is the most appropriate method for fitting presence‐only SDMs (Warton & Shepherd, 2010).The complementary log‐log (cloglog) transform was selected as a continuous index of environmental suitability, with 0 = low suitability and 1 = high suitability. Phillips et al. (2017) demonstrated the cloglog transform is equivalent to an IPP and can be interpreted as a measure of relative occurrence probability proportional to a species relative abundance.

We randomly selected 10,000 background absences recommended for regression‐based modeling (Barbet‐Massin et al., 2012) and to sufficiently sample the background calibration environment (Guevara et al., 2018). Convergent threshold was set at 10^–5^ and iterations increased to 5,000 from the default (500) allowing for model convergence. Optimal‐model selection was based on Akaike's information criterion (Akaike, 1974) corrected for small sample sizes (AIC_c_; Hurvich & Tsai, 1989), to determine the most parsimonious model by tuning two key MAXENT parameters: regularization multiplier and feature classes (Warren & Seifert, 2011). Eighteen candidate models of varying complexity were built by comparing a range of regularization multipliers from 1 to 5 in 0.5 increments, and two feature classes (Linear and Quadratic) in all possible combinations using the “checkerboard2” method of cross‐validation (*k*‐folds* = *5) within the ENMeval package in R (Muscarella et al., 2014). Response curves, parameter estimates, percent contribution, permutation importance, and a jackknife test were used to measure variable performance within the best‐fit model (see Appendix 1 in Supporting Information).

### 2.4 Model evaluation

Optimal‐model selection was evaluated using area under the curve (AUC) and omission rates. AUC is a nonparametric, threshold‐independent measure with AUC = 1.0 indicating maximum predictive performance, and AUC = 0.5 being no better than a random prediction. AUC_DIFF_ (AUC_TRAIN_ − AUC_TEST_) was used to quantify model overfitting (Muscarella et al., 2014), with a value close to zero indicating a low overfit model (Warren & Seifert, 2011). AUC metrics were used as a measure of optimal‐model selection, best suited to comparing a range of candidate models (Jiménez‐Valverde, 2012; Lobo et al., 2008). Omission rates are threshold‐dependent metrics for evaluating discriminatory ability and overfitting at specified thresholds. Lower omission rates show improved discrimination between suitable and unsuitable areas (indicating higher performance), while overfitted models show higher omission rates than expected by theory (Radosavljevic & Anderson, 2014). Omission rates were calculated based on two threshold rules: minimum training presence (MTP) and 10% training presence (10TP). For low overfit models, the expectation in MTP is a value close to zero and for 10TP a value close to 0.10.

Two further test metrics were used to evaluate the final best‐fit model. First, model accuracy was tested against random expectations using partial receiver operating characteristic (pROC), which estimates model performance by giving precedence to omission errors over commission errors (Peterson et al., 2008). Partial ROC ratios range from 0 to 2 with 1 indicating a random model. Function parameters were set with a 5% omission error rate, and 1,000 bootstrap replicates on 50% test data to determine significant (*α* = 0.05) pROC ratios > 1.0 in the R package ENMGadgets (Barve & Barve, 2013). Second, Continuous Boyce Index (CBI, Hirzel et al., 2006) was used to measure how much environmental suitability predictions differ from a random distribution of observed presences (Boyce et al., 2002). CBI is consistent with a Spearman correlation (*r_s_*) with values ranging from −1 to +1. Positive values indicate predictions consistent with observed presences, with values close to zero no different than a random model. Negative values indicate areas with frequent presences having low environmental suitability. Mean CBI evaluation was calculated using five‐fold cross‐validation on 20% test data with a moving window for threshold‐independence and 101 defined bins in the R package enmSdm (Smith, 2019).

### 2.5 Reclassified binary prediction

To calculate potential range size, the continuous current prediction was reclassified to a binary (suitable/unsuitable) prediction to complement the current IUCN geographic range polygon (BirdLife International, 2017). Currently, there is no consensus on choosing binary thresholds and threshold selection can be an arbitrary process (Liu et al., 2013, 2016). We selected 10% training presence (10TP), a threshold that removes the lowest 10% of predicted values accounting for any uncertainty in the occurrence data (Pearson et al., 2007), and visually best‐fitted current expert knowledge on harpy eagle distribution. We used the same 10TP threshold for the paleoclimate predictions because this provided a more realistic estimate for current range size to use for projecting into past climatic scenarios. Finally, we calculated extent of occurrence (EOO) with a minimum convex polygon around all our occurrence points (excluding the ocean) following IUCN guidelines (IUCN, 2019). General model development and spatial analysis were performed in R (v3.5.1; R Core Team, 2018) using the dismo (Hijmans et al., 2017), raster (Hijmans, 2017), rgdal (Bivand et al., 2019), rgeos (Bivand & Rundel, 2019), and sp (Bivand et al., 2013) packages.

### 2.6 Environmental ordination

To determine species–environment relationships in environmental space, the underlying environmental data at occurrence points were extracted using the three most important predictors from their contribution to model prediction. A random sample of 100,000 background points were extracted to represent the background environment, with occurrence data and environmental space defined using a minimum convex polygon. Ecological Niche Factor Analysis (ENFA, Basille et al., 2008; Hirzel et al., 2002) was calculated using all unfiltered occurrence points (*n* = 1,179), against the background environmental data. ENFA directly measures environmental conditions at the presence points; thus, spatial auto‐correlation in occurrence data is not considered a serious issue (Basille et al., 2008). Including as many presence points as possible is therefore advisable in ENFA to obtain accurate measures of occupied environmental space (Hirzel et al., 2001).

ENFA is a multivariate, factorial analysis extracting two measures of a species realized niche along two axes. The first axis metric, marginality (*M*), measures the position of the species ecological niche, and its departure relative to the available environment. A value of *M* > 1 indicates that the niche deviates more relative to the reference environmental background and has specific environmental preferences compared to the available environment. The second axis metric, specialization (*S*), is an indication of niche breadth size relative to the environmental background, with a value of *S* > 1 indicating higher niche specialization (narrower niche breadth). A high specialization value indicates a high reliance on the environmental conditions that mainly explain that specific dimension. ENFA was calculated in the R package CENFA (Rinnan, 2018), using a corrected calculation on the coefficient matrix for specialization and weighting all cells by the number of observations (Rinnan & Lawler, 2019). Predictors were rescaled; thus, the resulting ENFA can be interpreted similar to a PCA with eigenvalues and loadings represented along the first axis of marginality and the following secondary orthogonal axes of specialization (Basille et al., 2008).

## 3 RESULTS

### 3.1 Species distribution models

**Figure 1:**
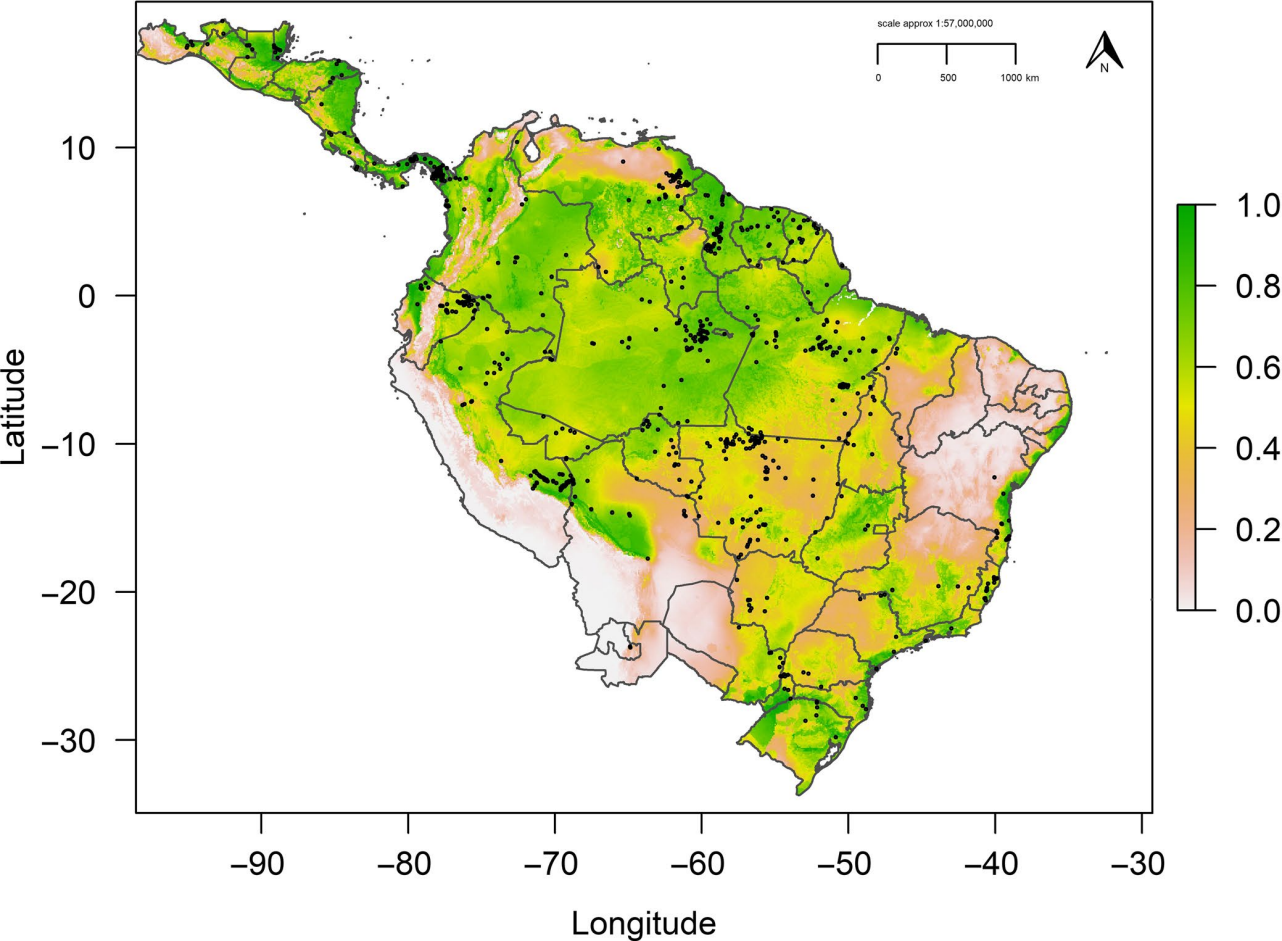
Predicted current distribution for the harpy eagle with values closer to 1 having highest environmental suitability. Gray borders represent national borders and internal state boundaries for Argentina, Brazil, and Mexico. Black points define harpy eagle occurrences

**Figure 2:**
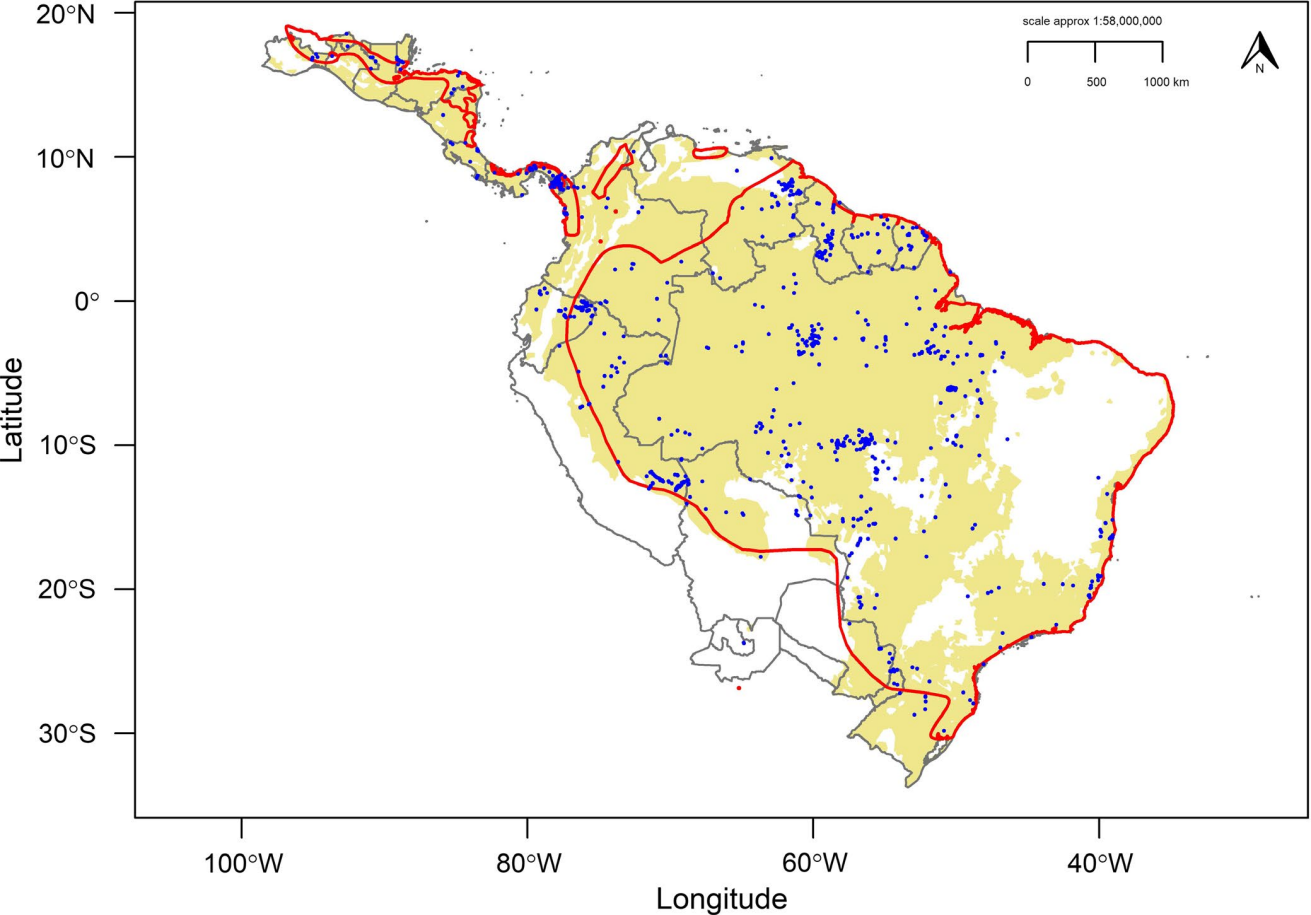
Reclassified binary range prediction for the harpy eagle using 10% training presence (10TP = 0.415) threshold. Khaki area is the suitable environmental space above the 10TP threshold, white areas not suitable. Red polygons define current IUCN range for the harpy eagle. Gray borders represent national borders and internal state boundaries for Argentina, Brazil, and Mexico. Blue points define harpy eagle occurrences

The best‐fit model (ΔAIC_c_ = 0.0) had feature classes linear and quadratic with a regularization multiplier of β = 1. AUC metrics showed moderate predictive performance (AUC_TRAIN_ = 0.698, AUC_TEST_ = 0.692), with minimal overfitting (AUC_DIFF_ = 0.06) and high discrimination ability with omission rates close to expected values (MTP = 0.003, 10TP = 0.11). Testing the model against random expectations resulted in robust mean pROC ratios (pROC = 1.407, *SD* ± 0.057, range = 1.235–1.577), with high calibration accuracy between predicted environmental suitability and test occurrence points (Mean CBI = 0.838). The continuous best‐fit model defined the spatial complexity in distribution for the harpy eagle and identified an area of highest abiotic suitability across Amazonia (Figure 1), with patchier distribution across southern Brazil and north into Central America (Figure S3, see Appendix 3). Reclassifying the continuous prediction using the 10TP threshold (0.415; Figure 2) gave an estimate for geographic range size of 9,844,399 km^2^. Based on our occurrence data, we estimated an EOO of 13,050,940 km^2^.

### 3.2 Environmental predictors

From parameter estimates, the harpy eagle was more likely to be associated with CMI and minimum temperature of the warmest month (Table 2). Overall, three predictors contributed 96% to model prediction. Climatic Moisture Index (CMI) contributed the highest percentage (72.1%, Table 3), with minimum temperature in the warmest month (15.6%) and Terrain Roughness Index (TRI, 8.3%) the next two highest contributions (Table 3). CMI had the highest regularized training gain, followed by precipitation in the wettest month and minimum temperature in the warmest month (Figure S4, see Appendix 3). CMI had the highest gain when used in isolation, so had the most useful information on suitable environmental conditions when used alone. CMI decreased the gain the most when omitted and could best explain the environmental requirements of the harpy eagle not present in the other predictors.

**Table 2:** Parameter estimates derived from beta‐coefficients for the harpy eagle distribution model fitted using linear and quadratic feature classes

Predictor	Linear	Quadratic
Climatic Moisture Index	1.38	−3.62
Minimum temperature warmest month	0.13	*
Maximum temperature warmest month	0.05	*
PET driest quarter	0.03	0.00
Precipitation wettest month	0.02	*
Terrain Roughness Index	0.02	0.00
Precipitation warmest quarter	0.00	*
Isothermality^2^	*	−0.01
PET wettest quarter^2^	*	0.00

**Table 3:** Percent contribution and permutation importance for variables used as environmental predictors in the current distribution model for the harpy eagle. All values are %

Predictor	Percent contribution	Permutation importance
Climatic Moisture Index^a^	72.1	43.1
Minimum temperature warmest month	15.6	22.8
Terrain Roughness Index^b^	8.3	12.4
PET driest quarter	3.0	9.8
PET wettest quarter	0.5	5.2
Isothermality^c^	0.2	5.2
Precipitation wettest month	0.2	5.2
Precipitation warmest quarter	0.0	0.7
Maximum temperature warmest month	0.0	0.4

^a^ Ratio of annual precipitation to annual evapotranspiration

^b^ Variation in local terrain around a central pixel

^c^ Mean diurnal temperature range/temperature annual range*100.

**Figure 3:**
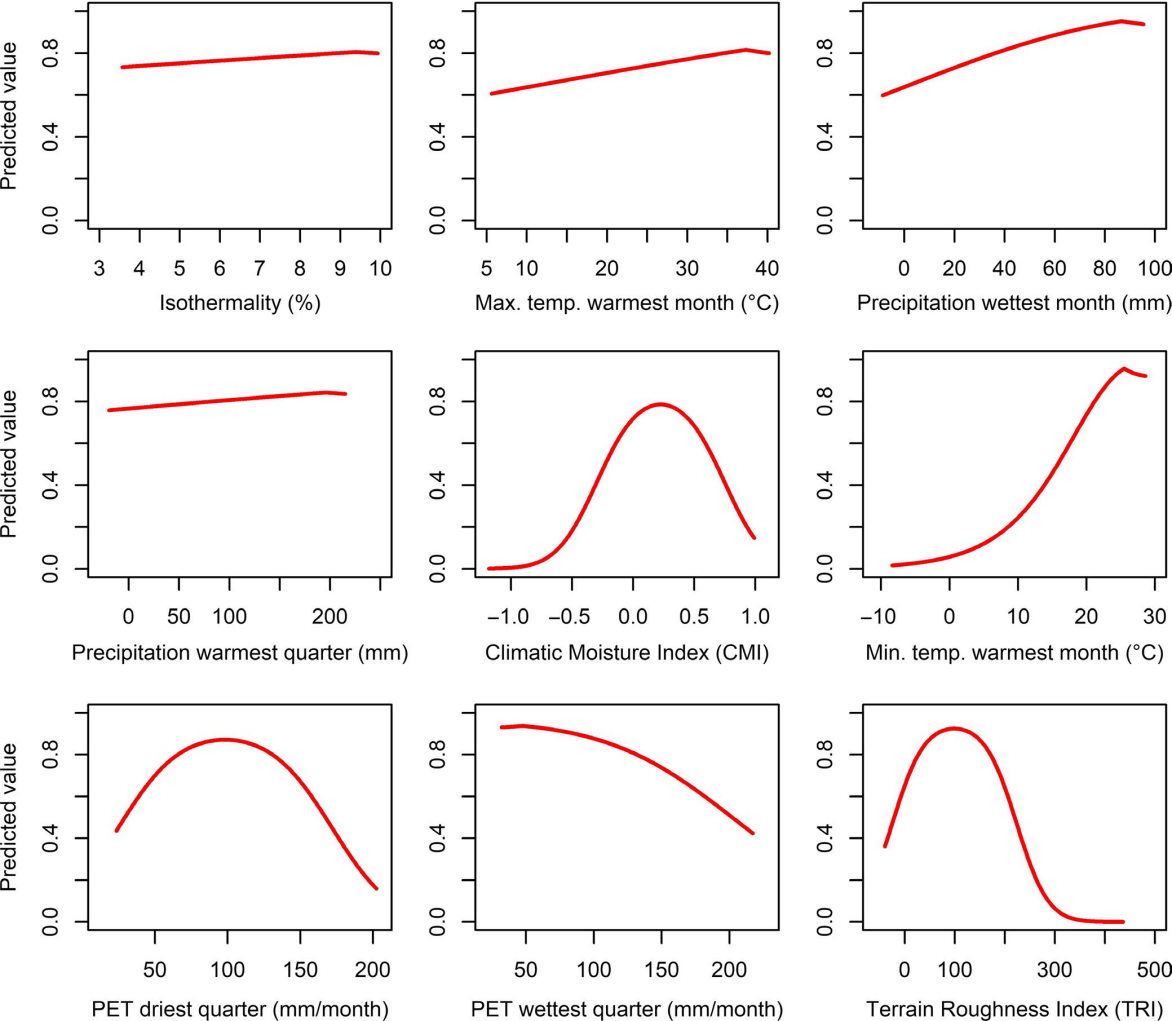
Response curves for predictors used in the current distribution model for the harpy eagle

From the response curves, there was a positive response to CMI peaking at ~0.4, with highest suitability for the minimum temperature of the warmest month increasing rapidly after 10°C, peaking at 25°C (Figure 3). Precipitation in the wettest month peaked at 90 mm/month, before leveling off up to 100 mm, with highest suitability for precipitation in the warmest quarter at 200 mm. Isothermality peaked at 9%–10%, reflecting the constant temperatures harpy eagles need in lowland tropical forests. PET in the driest quarter had highest suitability at 100 mm/month, but with highest suitability for PET in the wettest quarter at 50 mm/month indicating a preference for climates with greener vegetation. TRI peaked at 100 indicating high preference, as expected, for lowland flat areas with low terrain complexity.

### 3.3 Environmental ordination

**Figure 4:**
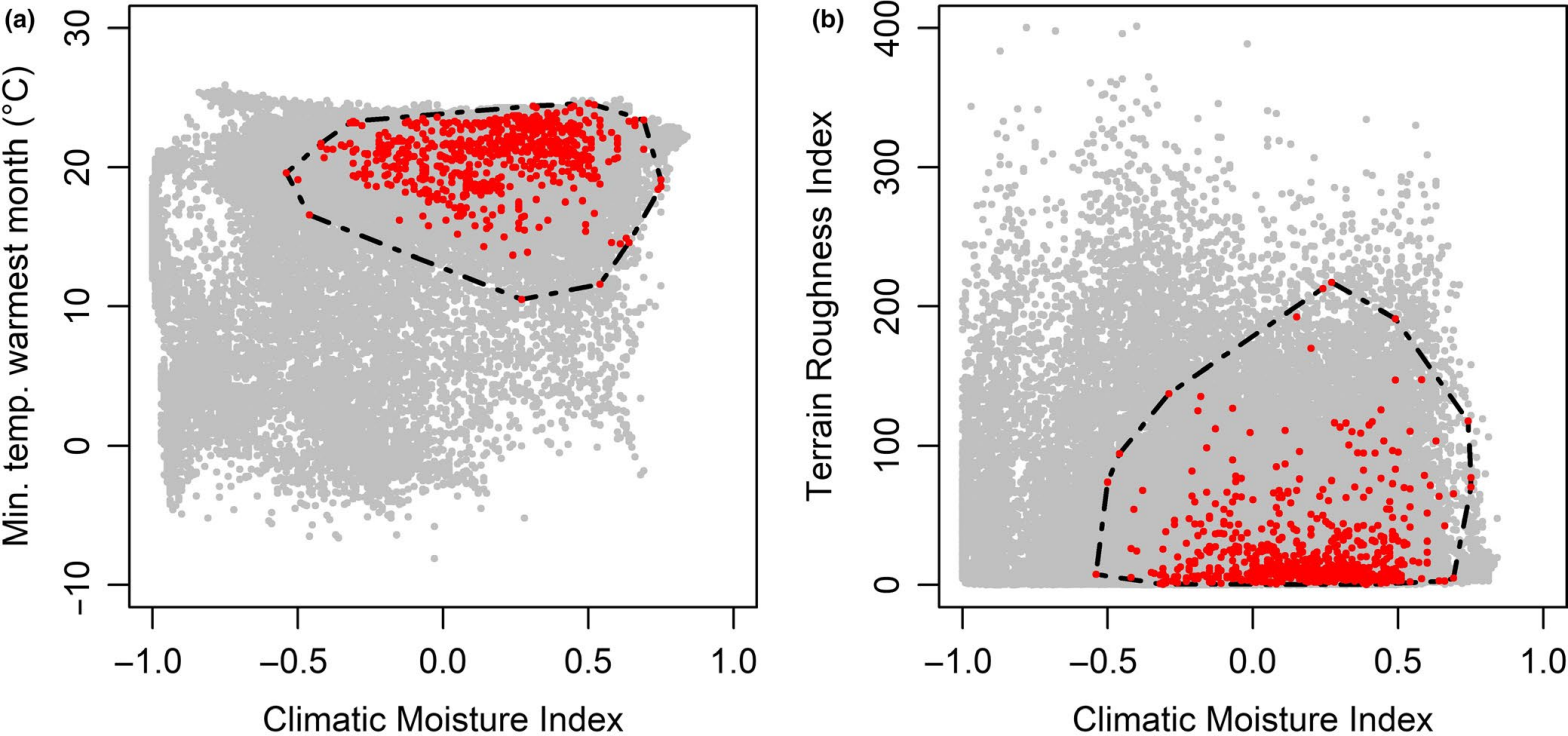
Distribution of harpy eagle occurrences in selected pairs of environmental variables. Gray points are random background environmental points, and red points are harpy eagle occurrences. Black hashed line defines the minimum convex polygon of harpy eagle occurrences

**Figure 5:**
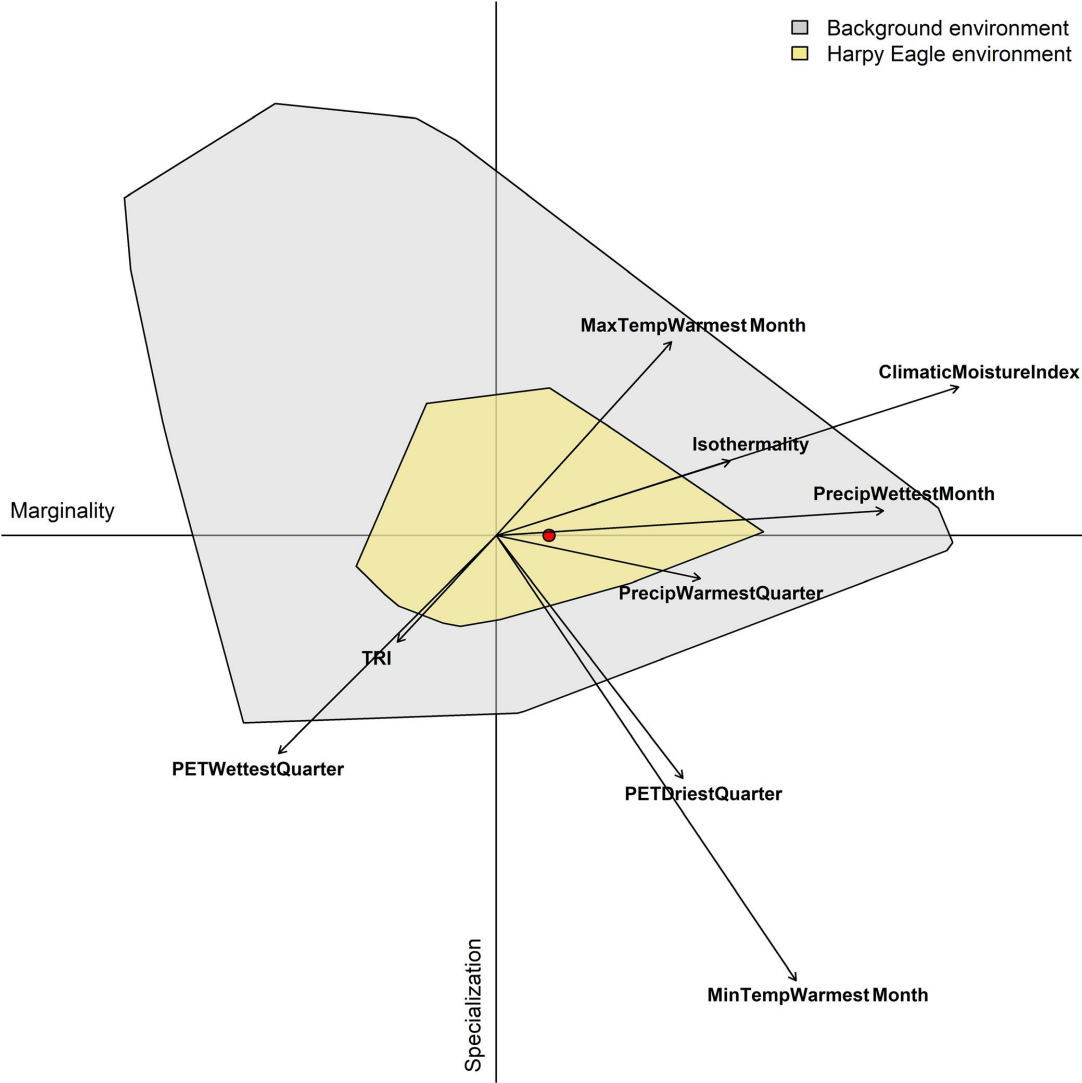
Ecological Niche Factor Analysis (ENFA) for suitable harpy eagle environment space (khaki) within the available background environment (gray) shown across the marginality (x) and specialization (y) axes. Arrow length indicates the magnitude with which each variable accounts for the variance on each of the two axes. Red circle indicates niche position (median marginality) relative to the average background environment (the plot origin)

Within selected axes of environmental space, harpy eagle occurrences were clustered within a Climatic Moisture Index ranging between −0.5 and 0.7 (Figure 4a). Harpy eagle occurrences showed a lower limit for minimum temperature with no location points below 10.5°C in the warmest month. Most occurrences were clustered around or above 20°C (Figure 4a), linked to the harpy eagle's preference for generally flat, lowland areas with low terrain complexity (Figure 4b). Harpy eagle environmental space did not deviate substantially from the average background environment available, with the ENFA marginality factor slightly below the available background environment (*M = *0.99; Figure 5, red circle). However, the harpy eagle is restricted to a particular environmental space relative to the reference environmental background with a narrow environmental niche breadth indicating highly specialized environmental requirements (*S* = 1.431). Five significant ENFA factors explained 80.75% of the total variance in niche structure, with the first specialization axis (Spec1) explaining 28.81% of this total (Table 4). CMI and precipitation in the wettest month were the two highest coefficients on the marginality axis, with minimum temperature in the warmest month the highest on the specialization axis.

**Table 4:** Variance explained by the five most significant factors (Marg. = marginality; Spec = Specialization) in an Ecological Niche Factor Analysis (ENFA) for suitable harpy eagle environment space

ENFA axis	Marg	Spec1	Spec2	Spec3	Spec4
Variance explained (%)	14.05	28.81	13.82	12.51	11.56
Predictor					
Climatic Moisture Index	0.56	0.24	−0.08	−0.24	0.26
Precipitation wettest month	0.47	0.04	0.00	−0.05	−0.04
Min. temp. warmest month	0.36	−0.72	−0.30	−0.28	−0.27
Isothermality	0.28	0.12	0.03	0.08	0.33
PET wettest quarter	−0.26	−0.35	−0.31	−0.40	0.20
Precipitation warmest quarter	0.25	−0.07	0.01	0.15	−0.15
PET driest quarter	0.23	−0.39	−0.49	−0.19	−0.56
Max. temp. warmest month	0.21	0.31	0.73	0.77	0.57
Terrain Roughness Index	−0.12	−0.17	−0.18	0.23	0.21

Note

Coefficient values for the nine environmental predictors are ordered according to the highest coefficient values in the marginality factor.

### 3.4 Paleo‐distributions

**Figure 6:**
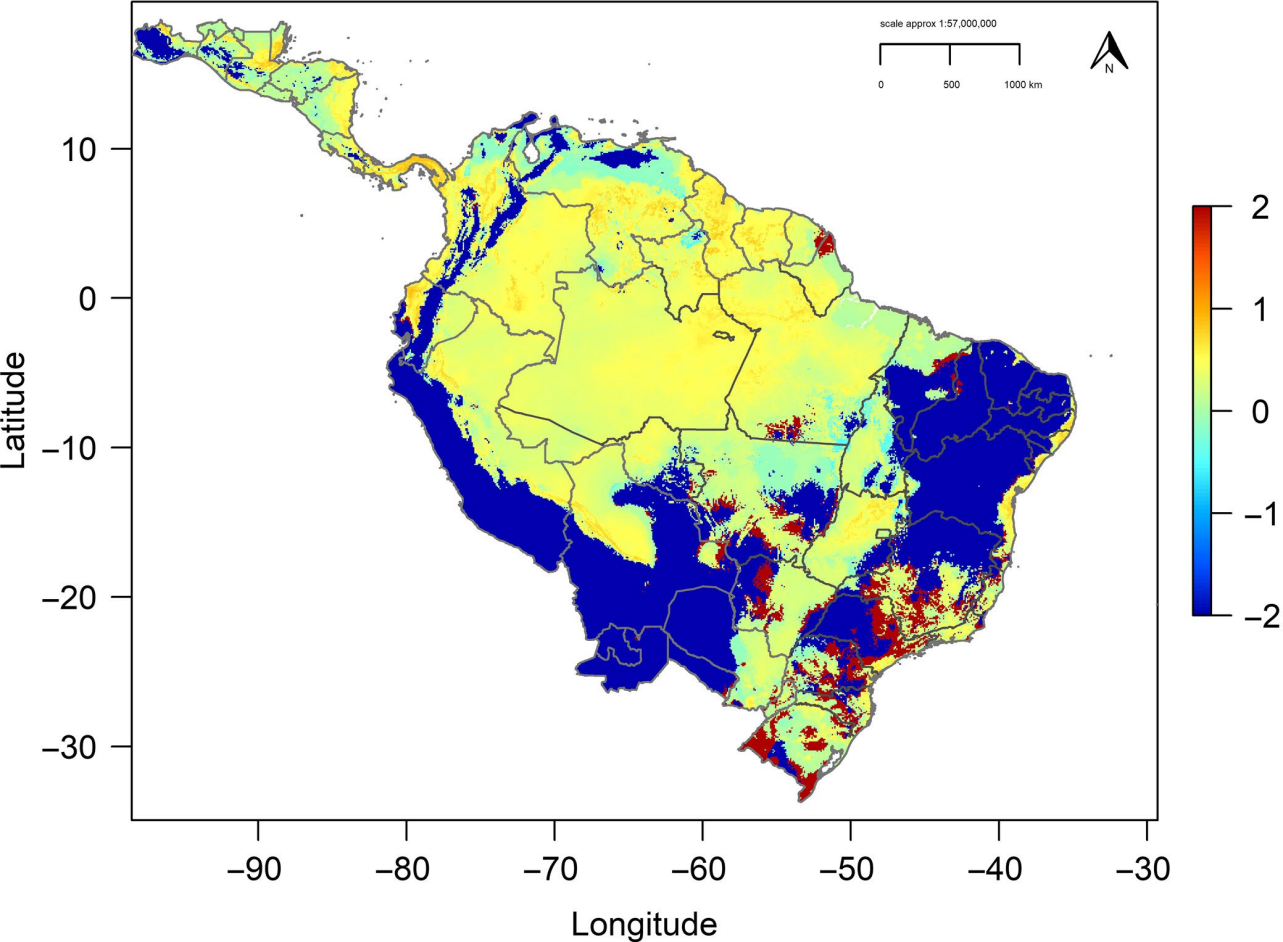
Predicted climate stability for the harpy eagle summed from the current, Last Glacial Maximum (LGM, ~22,000 years ago) and Mid‐Holocene (~6,000 years ago) predictions. Values of −2 indicate species absence, −1 to 0 shows colonizable areas, 0 to 1 defines areas of highest stability, and values of 2 (dark red patches) show the most unstable areas. Map defines summed prediction masked to current geographic extent and geo‐political boundaries

All individual paleoclimate GCMs predicted similar paleo‐distributions with high geographic niche overlap (Table S1, see Appendix 2 in Supplementary Information; Figures S5 and S6, see Appendix 3). From the mean projections, hindcasting the current prediction to the LGM defined a large area of high suitability across northern‐central South America. A further strip of high suitability extended from present‐day Panama, south along the Pacific slope west of the Andes into the present‐day Chocó region and west Ecuador (Figure S7, top left). In the Mid‐Holocene, high suitability areas increased, extending north into Central America, across Amazonia and east in present‐day Brazil (Figure S7, top right). During the LGM, mean range size was 17% smaller (Figure S7, bottom left; Table S2, see Appendix 2), compared to the current 10TP geographic range size (9,844,399 km^2^). In the Mid‐Holocene, range size had increased from the LGM, but was still 6% smaller than the current 10TP range size estimate (Table S2, see Appendix 2; Figure S7, bottom right). Areas of highest stable refugia were identified in the central Amazon basin north into Guyana, south‐east Colombia, and Panama (Figure 6), consistent with these areas having continuous high suitability since the LGM.

## 4 DISCUSSION

More than half of all global raptor species have declining populations, and there is a significant knowledge gap on the extent of their distribution and ecological requirements (McClure et al., 2018). In particular, accurate distribution estimates are lacking for many tropical forest raptors (Buechley et al., 2019; Sarasola et al., 2018). We provide an analytical framework for applying predictive spatial models to address these fundamental issues to a tropical forest raptor. More broadly, we propose this analytical framework as an efficient and cost‐effective approach to tackling this problem across all taxa. Using a PPM regression framework is now viewed as one of the most effective methods to determine species distributions and relative abundance (Aarts et al., 2012; Isaac et al., 2019; Renner et al., 2015), as supported by our results. Using climatic and topographical predictors resulted in high model predictive performance, defining in more detail the spatial and environmental requirements for the harpy eagle across its geographic range. However, we recognize that including predictors such as landcover and human impact, which are changing rapidly, would improve predictions. These, however, will be analyzed and presented elsewhere.

### 4.1 Spatial requirements

How species are distributed in geographic and environmental space is fundamental to conservation planning (Loiselle et al., 2003; Pearce & Boyce, 2006). Yet accurate and reliable spatial information, such as geographic range size and environmental constraints, is often lacking in many tropical biodiversity assessments (Cayuela et al., 2009; Tobias et al., 2013), and specifically for Neotropical raptors (Sarasola et al., 2018). Using a PPM framework enables the predictions given here to be interpreted as areas of relative abundance (Phillips et al., 2017; Renner et al., 2015) under the assumption that historical habitat is still intact. Building on a previous SDM (Miranda et al., 2019), our continuous prediction adds further spatial detail showing a discontinuous distribution. This is likely a consequence of patchy environments, resulting in spatial heterogeneity in harpy eagle distribution. Miranda et al. (2019) used both climatic and vegetation predictors, and there is a close visual correspondence between their predictions and both our continuous and binary models. This suggests that at the continental scale, biologically relevant climatic and topographical predictors alone can accurately predict the distribution for the harpy eagle.

Our models refine previous coarse estimates of harpy eagle distribution (Birdlife International, 2017; Ferguson‐Lees & Christie, 2005), providing an empirically derived range size to complement the species’ current IUCN status. Our binary threshold polygon estimate of geographic range size (Figure 2; 9,844,399 km^2^) was 11% smaller than the current IUCN polygon (11,064,295 km^2^), and our estimated EOO (13,050,940 km^2^) was 25.9% less than the current IUCN EOO (17,600,000 km^2^). Based on these figures, we recommend reviewing the IUCN distributional area for the harpy eagle, which can overestimate avian geographic range sizes (Jetz et al., 2007; Peterson et al., 2016; Ramesh et al., 2017). Specifically, the removal of semiarid areas (such as the Caatinga in eastern Brazil) from across the IUCN range would show a more realistic geographic distribution. The Caatinga area had low predicted suitability, no current or historical occurrence records, and was not predicted suitable for the harpy eagle including during the last glacial maximum (LGM). Similarly, the Cerrado (in central Brazil) was not predicted as suitable for the harpy eagle either during the LGM, and all recent records for the species show no evidence of breeding in the area. Although early naturalists reported breeding harpy eagles in this region (Sick & Barruel, 1984), there is no evidence of a functional population and the area should be removed from the IUCN range polygon (and any present range projections) following IUCN guidelines for not including areas where the species does not exist (IUCN, 2019).

### 4.2 Species–environment relationships

The continuous model highlighted distinct areas of high environmental suitability (Figure 1), with the binary model closely matching the primary vegetation types for recognized harpy eagle habitat (lowland tropical broadleaf forest, Beck et al., 2018). Thus, in the Chocó biogeographic region of north‐west Ecuador and south‐west Colombia west of the Andes, the current model defined areas of high environmental suitability, which correlate with new records of harpy eagles in the Pacific slope region (Muñiz‐López, 2005; Muñiz‐López et al., 2007; Zuluaga et al., 2018). However, due to continued habitat loss in this area and across the species range, climatically suitable areas predicted for some regions may over‐represent suitability where there is no longer harpy eagle forest habitat. Our models also defined previously unrecognized areas of high environmental suitability in south‐east Colombia, northern Guyana, and along the east Andean slope of Peru and Bolivia. All these regions may hold viable populations of harpy eagles, with further research and continued surveys in these areas recommended where possible.

Environmental suitability predicted for the harpy eagle largely correlates with habitat selection studies from Amazonian Peru (Robinson, 1994). Here, highest frequency of harpy eagle sightings was recorded in mature flood plain forest, with high nesting densities below 300 m elevation in lowland humid forest in Darien, Panama (Vargas González & Vargas, 2011), analogous to the environmental suitability predictions here. Due to the rarity and large home range sizes of harpy eagles, Thiollay (1989) was not able to provide population density estimates from French Guiana, but suggested harpy eagles are rare but widespread throughout the largely tropical lowland forest in the region, consistent with our results. Although largely thought to be extirpated from much of Central America, our models identify areas of high suitability for harpy eagles along the Caribbean slopes of Costa Rica, Honduras, Nicaragua, and Panama (Figure S3), which should be prioritized for continued surveys and habitat protection.

Using the combined analytical approach enabled a further development of the spatial modeling process by unraveling the preferred environmental space and ecological conditions where harpy eagle abundance should be at its highest (Osorio‐Olvera et al., 2019; VanDerWal et al., 2009). Climatic Moisture Index (CMI) was the most important environmental variable defining harpy eagle distribution, with a preferred CMI = ~0.4 (Figure 3), along with the highest model gain when used solely in a jackknife test, demonstrating its importance to account for harpy eagle distribution. This indicates a preference for wet, moist environments, correlating with lowland tropical forest across Central and South America (Beck et al., 2018; Willmott & Feddema, 1992), and suggests that CMI may be a useful surrogate predictor for habitat in tropical areas. Aligned with CMI and lowland tropical forest distribution was the positive response to higher minimum temperatures in the warmest month (Figure 3). Harpy eagle environmental suitability was highest in areas with a minimum temperature of ~24°C, reflected in the stable temperature conditions found across lowland tropical forests.

Assessing harpy eagle distribution in environmental space revealed similar patterns of environmental tolerances to the geographic models (Figures 4 and 5), with CMI having the highest positive correlation with harpy eagle occurrence. However, precipitation in the wettest month was also highly correlated with harpy eagle occurrence (Table 4), following the general observation for tropical regions that seasonal rainfall patterns are the main limiting factor for primary productivity and therefore species distributions (Schloss et al., 1999; Williams & Middleton, 2008). The ENFA confirmed the specialized environmental requirements for the harpy eagle, strongly linked to CMI and precipitation, which are likely operating as useful surrogate predictors of lowland tropical forest habitat. Importantly, minimum temperature of the warmest month (MTWM) had a high negative coefficient value on the specialization axis (Table 4). This indicates that MTWM is a key climatic predictor restricting harpy eagle distribution, linked to harpy eagle preference for lower elevations (Muñiz‐López, 2008; Piana, 2007; Vargas González & Vargas, 2011). Harpy eagle nests are rarely found above an altitude of 300 m (Vargas González & Vargas, 2011), and as temperature and elevation are closely correlated it seems likely the harpy eagle is negatively responding to lower temperatures at higher elevations restricting breeding distribution.

### 4.3 Paleo‐distributions

The two paleoclimate predictions given here place current harpy eagle distribution in context. During the LGM, highest suitability was centered on northern and western Amazonia and present‐day Panama. This follows current evidence that suggests during the LGM much of Amazonia was forested (Mayle et al., 2004), contrary to the rainforest refugia hypothesis (Haffer, 1969). However, forest structure was likely quite different from the present‐day, due to lower temperatures, rainfall, and atmospheric CO_2_ (Mayle et al., 2004), resulting in mixed‐forest communities. Climate reconstructions from Amazonia during the LGM show that temperatures were 5°C cooler than today (Guilderson et al., 1994; Stute et al., 1995) and that rainfall was spatially highly variable, as it is in the present‐day. Thus, dry forest‐savannahs may have dominated the region of central and southern Amazonia during the LGM, which may explain the low environmental suitability for the harpy eagle in this region from the LGM paleoclimate model.

During the Mid‐Holocene, the continuous prediction was similar to the current model with expansion of high suitability across Amazonia and north into Central America (Figure S7, top right, Appendix 3). This may be explained by the correlation of these areas with expansion of deciduous broadleaf forest in the region during the Mid‐Holocene, ultimately related to changing precipitation levels (Mayle et al., 2004). The increase in distributional area size during this period correlates with a population expansion identified from genetics from 60,000 cal yr BP, well before the LGM, and subsequently through the Mid‐Holocene (Lerner et al., 2009). The population expansion prior to the LGM occurred with climatic changes in Amazonia, leading to a reduction of tropical forest (Mayle et al., 2004), followed by expansion of forest through the LGM and Mid‐Holocene up to pre‐Industrial times. Thus, harpy eagle distribution area is strongly associated with changing climatic conditions (and therefore vegetation), which suggests a potential reduction in range size under future drier climate change conditions predicted across much of Central and South America (da Costa et al., 2010). However, our stable refugia prediction identified key areas of stable conditions since the LGM where a suitable climatic envelope for the harpy eagle is likely to persist into the future (Figure 6). We recommend these areas be prioritized for conservation and research, holding some encouragement for the future survival of the species as long as habitat can be maintained.

Explaining the observed distribution and ecological constraints of an organism by reference to its environmental requirements is one of the central goals in ecology (Krebs, 2009). Species at high trophic levels with slow life histories are often at increased risk of extinction (Purvis et al., 2000). Therefore, understanding the environmental processes regulating distribution of apex predators is an especially pressing conservation need. By refining previous range estimates using relevant abiotic variables (including those that may act as vegetation surrogates), our models define the ecological processes shaping both current and past harpy eagle distribution. However, future distribution models should include variables such as biotic interactions, landcover and human impacts at broad and fine scales to improve current predictions, and project into future climate change scenarios. With recent work demonstrating strong relationships between suitability predictions from SDMs and species abundance (Osorio‐Olvera et al., 2020; Weber et al., 2017), we confirmed the suitability of spatial point process models to deliver cost‐effective and reliable first estimates of relative abundance for species conservation management. Having accurate distributional data on the current ranges of tropical birds and raptors has long been a priority in the Neotropics (Bierregaard, 1998; Snow, 1985). Using a range of spatial modeling methods, we were able to establish a baseline of ecological constraints for the harpy eagle that may help to better plan its conservation across its vast continental distribution.

## CONFLICT OF INTEREST

None declared.

## AUTHOR CONTRIBUTIONS

**Luke J. Sutton:** Conceptualization (lead); data curation (equal); formal analysis (lead); investigation (lead); methodology (lead); visualization (lead); writing –original draft (lead); writing–review and editing (lead). **David L. Anderson:** Data curation (equal); validation (equal); writing–review and editing (supporting). **Miguel Franco:** Conceptualization (supporting); formal analysis (supporting); investigation (supporting); methodology (supporting); project administration (supporting); supervision (equal); writing–review and editing (equal). **Christopher J. W. McClure:** Conceptualization (supporting); data curation (equal); formal analysis (supporting); investigation (supporting); methodology (supporting); project administration (supporting); supervision (equal); writing–review and editing (equal). **Everton B. P. Miranda:** Data curation (equal); validation (equal); writing–review and editing (supporting). **F. Hernán Vargas:** Data curation (equal); validation (equal); writing–review and editing (supporting). **Jose de J. Vargas Gonzalez:** Data curation (equal); validation (equal); writing–review and editing (supporting). **Robert Puschendorf:** Conceptualization (supporting); formal analysis (supporting); investigation (supporting); methodology (supporting); project administration (equal); supervision (equal); writing–review and editing (equal).

## Supporting information

Supplementary Material

## Data Availability

The data that support the findings of this study are openly available on the data repository *Dryad*: https://doi.org/10.5061/dryad.9cnp5hqgn
