# Supplementary material for: Geographic range estimates and environmental requirements for the harpy eagle derived from spatial models of current and past distribution
Source: Ecol Evol. 2020 Dec 15;11(1):481–97. doi: 10.1002/ece3.7068 (PMC7790654; doi:10.1002/ece3.7068)
Supplement: Supplementary file 1 — Supplementary Material [file ECE3-11-481-s001.docx]

**Supporting Information**

**Appendix 1**

**Methods**

**Harpy Eagle occurrence data**

Spatial auto-correlation in occurrence data was measured using Global Moran’s *I* index on an inverse Euclidean distance matrix projected into Lambert Azimuth Equal Area (LAEA) for South America using the R package ape (Paradis *et al.* 2004). Moran’s *I* is an index ranging from -1 to +1, with values closer to zero indicating no spatial auto-correlation, and values approaching the extremes indicating stronger negative or positive autocorrelation, respectively. Spatial clustering was measured using Nearest Neighbour Index (NNI) in the R package spatialEco (Evans 2018) with a convex hull window. NNI is the ratio of the observed distance divided by the expected distance between neighbours in a hypothetical random distribution. NNI < 1 indicates spatial clustering, with values of 1 indicating random dispersion, and those closer to 2 indicating regular dispersion. Cleaned occurrence data were spatially auto-correlated (Moran’s *I* = 0.808, *p* < 0.001) and showed spatial clustering (NNI = 0.333, *z* = -43.794, *p =* <0.001). After applying the 4km spatial filter, spatial auto-correlation was reduced (Moran’s *I* = 0.534, *p* < 0.001), with clustering moving towards random dispersion (NNI = 0.492, *z* = -26.471, *p* < 0.001) resulting in 742 spatially-filtered occurrence records for use in the calibration models (Fig. S2 in Appendix 3; mean elevation = 264.04 m, range = 2.00 - 2177.00 m).

**Environmental predictors**

Isothermality quantifies variation of annual temperatures using the size of oscillation between day-night temperatures relative to annual temperatures and is a useful predictor for tropical species (Nix 1986). Isothermal values < 100 indicate low levels of temperature variability within an average month relative to the year. Climatic Moisture Index (CMI) is a scaled measure (-1 ≤ CMI ≤ 1) of the ratio of annual precipitation and annual evapotranspiration (Willmott & Feddema 1992). Two variables of seasonal potential evapotranspiration (PET) were included as proxies for vegetation greenness, ultimately determined by ambient energy (Currie 1991). Terrain Roughness Index (TRI) is a measure of variation in topography around a central pixel, with lower values indicating flat terrain and higher values indicating larger differences in elevation of neighbouring pixels (Wilson *et al.* 2007). Terrain complexity and variability can be an important determinant of species distributions and may be viewed as a surrogate for habitat variability because rougher topography is often associated with variation in habitat (Riley *et al.* 1999). As the harpy eagle generally prefers flatter, lowland areas (Vargas-González & Vargas 2011), including TRI should be an important predictor with an expected harpy eagle preference for areas with lower TRI values.

**Species Distribution Models**

Only Linear and Quadratic features were considered to produce less complex and more realistic predictions (Merow *et al.* 2013; Guevara *et al.* 2018). Omitting Threshold and Product feature classes generally results in increased model performance and more biologically meaningful model interpretations (Phillips *et al.* 2017). Hinge features were used in calibration models, but resulted in biologically unrealistic response curves, and were therefore omitted. The checkerboard cross-validation method of partitioning masks the geographical structure of the data according to latitudinal and longitudinal lines, dividing all occurrences into four spatially independent bins of equal numbers. By masking the geographical structure of test data, the models are projected onto an evaluation region not included in the calibration process. All occurrence and background test points are assigned to their respective bins dependent on location, thus further reducing spatial auto-correlation between testing and training localities (Radosavljevic & Anderson 2014).

Percent contribution is the relative increase to model gain that each variable makes to the optimal solution arrived at by MAXENT. This contribution is dependent on the particular algorithm path chosen. On the other hand, permutation importance is independent of the algorithm path and represents the importance of a given value on the AUC training values (Phillips *et al.* 2006). Jack-knife tests exclude each value, then develop the model with a sole variable to determine percentage contribution and regularized training gain of each environmental variable to model performance. Training gain indicates how closely the model predicts the presence locations. The variables with the highest gain are those providing a good fit to the data and making greatest contribution to the final model. Whilst those variables decreasing the gain most when omitted are considered the most important explanatory variables (Phillips *et al.* 2006).

**Results**

**Species Distribution Models**

In Brazil, the states of Amazonas and Pará had highest suitability, with further high suitability in Acre and Roraima states. Guyana had a large area of high environmental suitability, extending east into both northern Suriname and French Guiana, and west into southern Venezuela. In Bolivia and Peru, high suitability was identified along the entire east Andean slope, extending north into Ecuador. High suitability was identified across eastern Colombia despite only patchy occurrence records in the region. In Ecuador, high suitability was highlighted both along the Pacific slope and east of the Andes. In central America, eastern Panama had very high environmental suitability, extending into west Panama, along the Carribbean Sea coast of Costa Rica, Nicaragua, eastern Honduras, and central-southern Belize (Fig. S3). Guatemala had disjunct high suitability, with a large area in the Peten Department conncected to central-southern Belize and a narrow strip along the Pacific coast (Fig. S3).

**References**

Currie, D.J. (1991). Energy and large-scale patterns of animal and plant-species richness. *The American Naturalist*. 137: 27-49.

Evans, J.S. (2018). *spatialEco.* R package version 0.1.1-1. [https://CRAN.R-project.org/package=spatialEco](https://cran.r-project.org/package=spatialEco).

Guevara, L., Gerstner, B.E., Kass, J.M., & Anderson, R.P. (2018). Toward ecologically realistic predictions of species distributions: A cross‐time example from tropical montane cloud forests. *Global Change Biology*. 24: 1511-1522.

Merow, C., Smith, M.J. & Silander, J.A. (2013) A practical guide to MaxEnt for modeling species’ distributions: what it does, and why inputs and settings matter. *Ecography.* 36: 1058-1069.

Nix, H.A. (1986). A biogeographic analysis of the Australian elapid snakes. In: Longmore, R. (Ed.). *Atlas of Elapid Snakes of Australia.* pp. 4–15. Australian Flora and Fauna Series No. 7.

Paradis, E., Claude, J. & Strimmer, K. (2004). ape: analyses of phylogenetics and evolution in R language. *Bioinformatics*. 20: 289-290.

Phillips, S.J., Anderson, R.P., Dudík, M., Schapire, R.E. & Blair, M.E. (2017). Opening the black box: an open‐source release of Maxent. *Ecography*. 40: 887-893.

Phillips, S.J., Anderson, R.P. & Schapire, R.E. (2006). Maximum entropy modeling of species geographic distributions. *Ecological Modelling*. 190: 231-259.

Radosavljevic, A. & Anderson, R.P. (2014). Making better Maxent models of species distributions: complexity, overfitting and evaluation. *Journal of Biogeography.* 41: 629-643.

Riley, S.J., DeGloria, S.D. & Elliot, R. (1999). Index that quantifies topographic heterogeneity. *Intermountain Journal of Sciences*. 5: 23-27.

Vargas-González, J. de J. & Vargas, F.H. (2011). Nesting density of Harpy Eagles in Darien with population size estimates for Panama. *Journal of Raptor Research*. 45: 199-211.

Willmott, C.J. & Feddema, J.J. (1992). A more rational climatic moisture index. *The Professional Geographer*. 44: 84-88.

Wilson, M.F., O’Connell, B., Brown, C., Guinan, J.C. & Grehan, A.J. (2007). Multiscale terrain analysis of multibeam bathymetry data for habitat mapping on the continental slope. *Marine Geodesy*. 30: 3-35.

**Appendix 2**

**Table S1.** Geographical niche overlap calculated using Schoener’s *D* for predicted paleo-distributions for the harpy eagle at the Last Glacial Maximum (~22,000ya) and Mid-Holocene (~6,000ya) using three paleoclimate General Circulation Models (GCMs).

| Last Glacial Maximum | |  |
| --- | --- | --- |
| GCM | CCSM4 | MIROC-ESM |
| MIROC-ESM | 0.836 |  |
| MPI-ESM-P | 0.841 | 0.814 |
|  |  |  |
| Mid-Holocene |  |  |
| GCM | CCSM4 | MIROC-ESM |
| MIROC-ESM | 0.871 |  |
| MPI-ESM-P | 0.896 | 0.869 |

**Table S2.** Geographic range size estimates from predicted paleo-distributions for the harpy eagle at the Last Glacial Maximum (~22,000ya) and Mid-Holocene (~6,000ya) using three paleoclimate General Circulation Models (GCMs). Area difference for each Paleoclimate GCM is compared to the reclassified 10TP current threshold prediction.

| Paleoclimate scenario | GCM | Area (km^2^) | Area difference (km^2^) | % |
| --- | --- | --- | --- | --- |
| Last Glacial Maximum | CCSM4 | 7,040,774 | 2,803,625 | -28.48 |
|  | MIROC-ESM | 9,422,859 | 421,540 | -4.28 |
|  | MPI-ESM-P | 7,914,406 | 1,929,993 | -19.60 |
|  | Mean | 8,126,013 | 1,139,386 | -17.45 |
|  |  |  |  |  |
| Mid-Holocene | CCSM4 | 9,292,721 | 551,678 | - 5.60 |
|  | MIROC-ESM | 9,436,390 | 408,009 | -4.14 |
|  | MPI-ESM-P | 9,013,362 | 831,037 | -8.44 |
|  | Mean | 9,247,491 | 596,908 | -6.06 |

**Appendix 3**

**
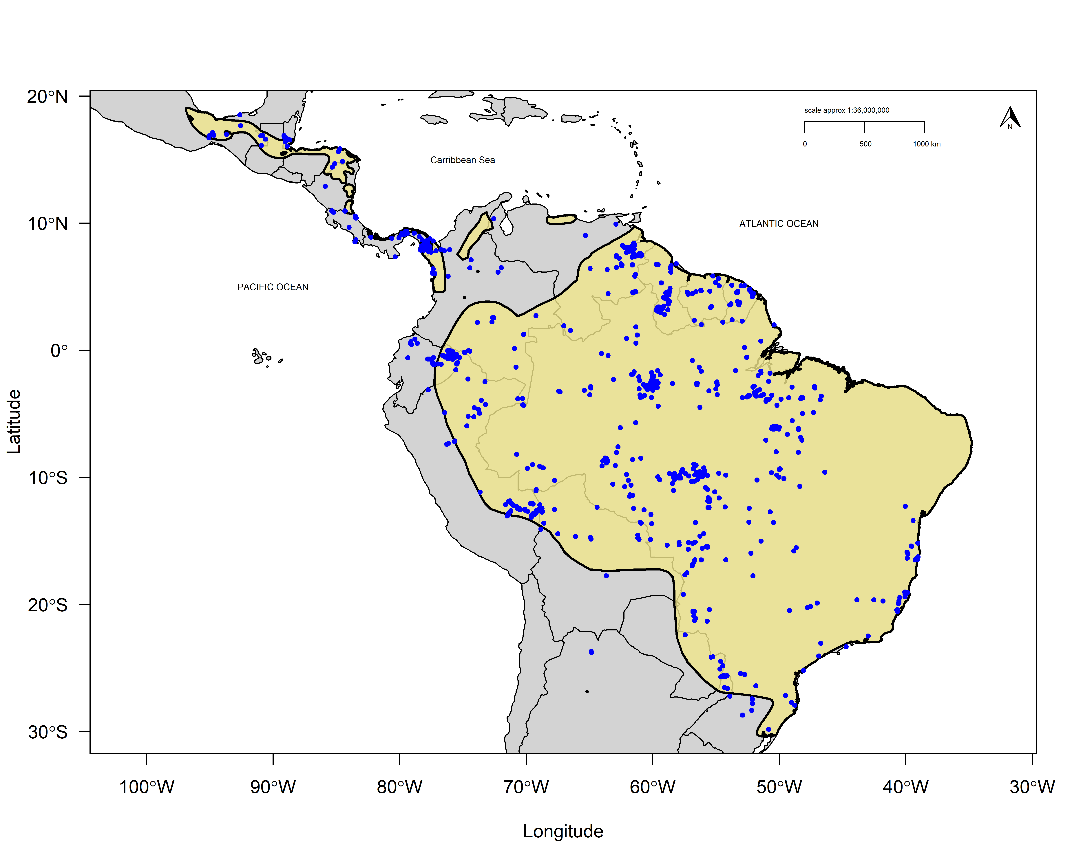
**

**Figure S1.** Current IUCN range map for the harpy eagle overlaid with unfiltered harpy eagle occurrence data (blue points) used in this study.

**
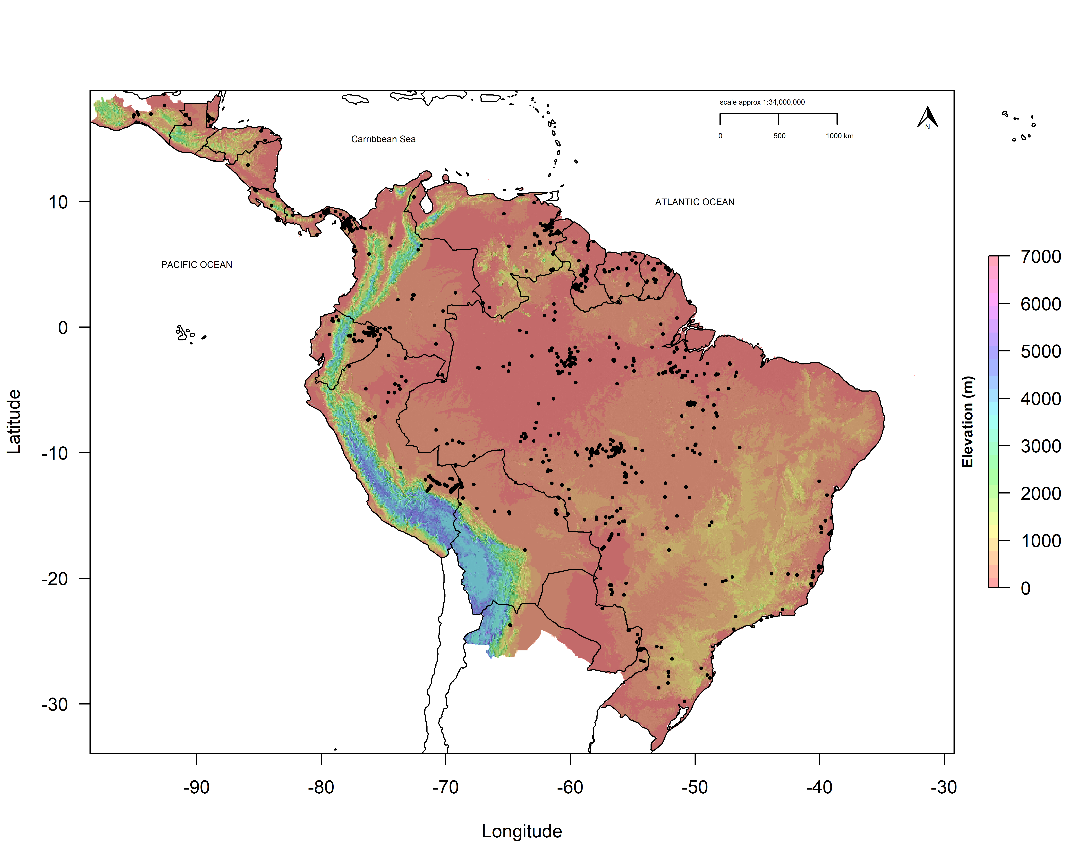
**

**Figure S2.** Digital Elevation Model showing distribution of harpy eagle occurrence data (black points) used in this study after applying a 4km spatial filter on the raw occurrence points.

**
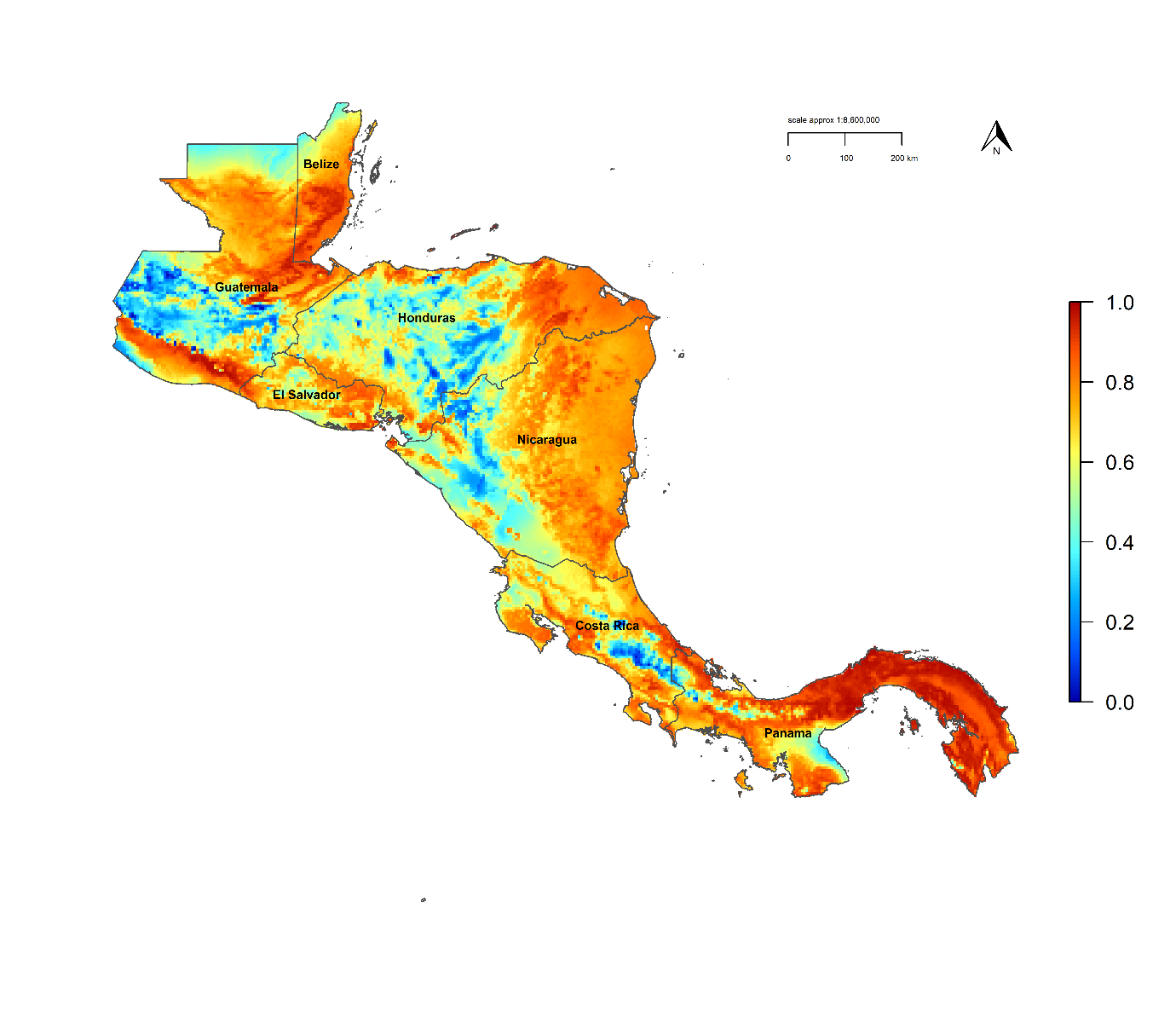
**

**Figure S3.** Projected distribution model for the harpy eagle for Central America. Map denotes complementary log log (cloglog) prediction with values closer to 1 defining higher environmental suitability for harpy eagle occurrence.

**Figure S4.** Variation in regularized training gain for climatic and topographical predictors using a jack-knife test of variable importance. Blue bars represent regularized gain without the variable and dark grey bars represent regularized gain with only the one variable.

**
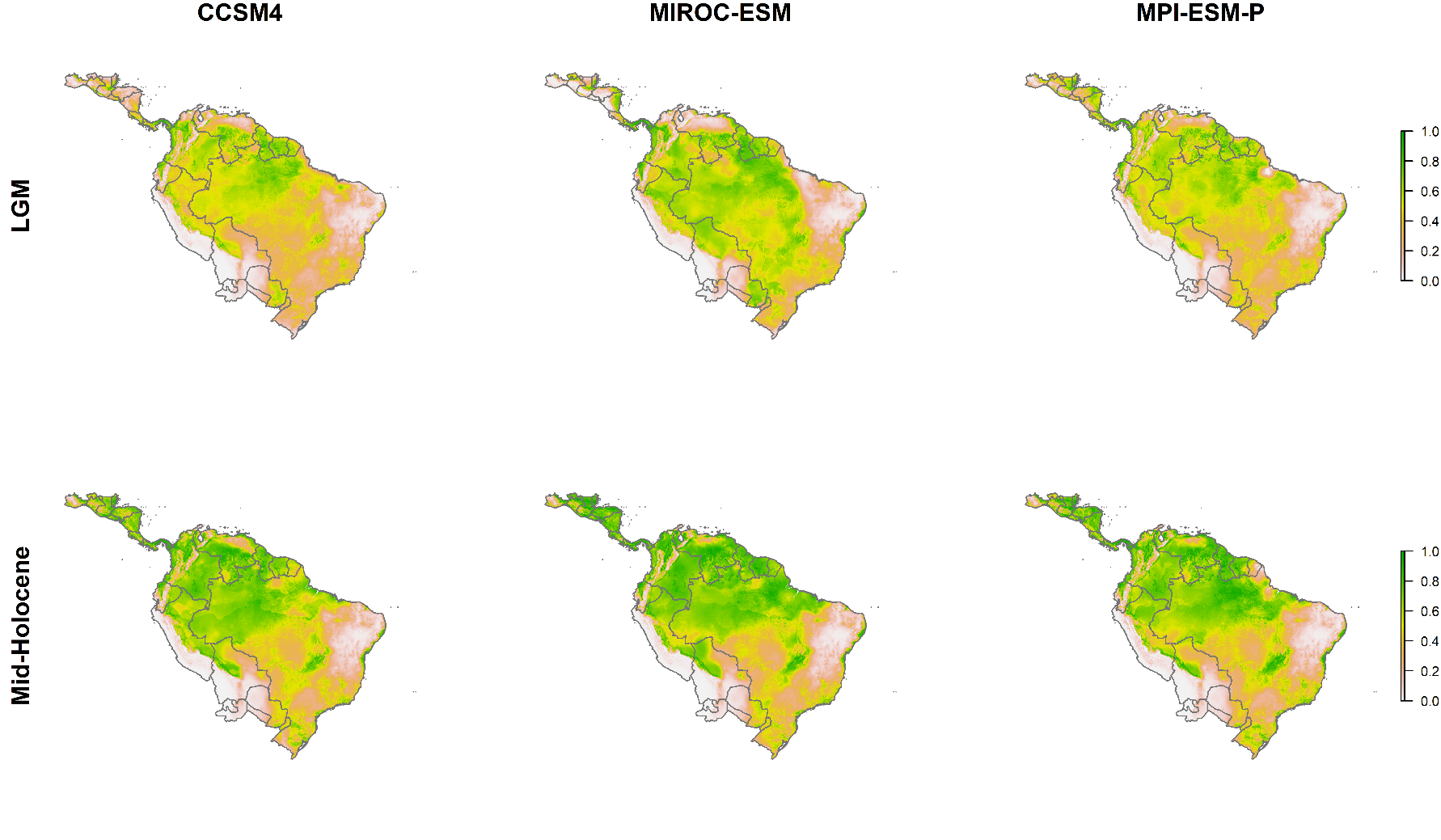
**

**Figure S5.** Predicted continuous Paleo-distributions for the harpy eagle projected to the Last Glacial Maximum (LGM, ~22,000ya) and Mid-Holocene (~6,000ya) across three paleoclimate General Circulation Models (GCMs). Maps denote complementary log log (cloglog) prediction with values closer to 1 defining higher environmental suitability for harpy eagle occurrence.

**
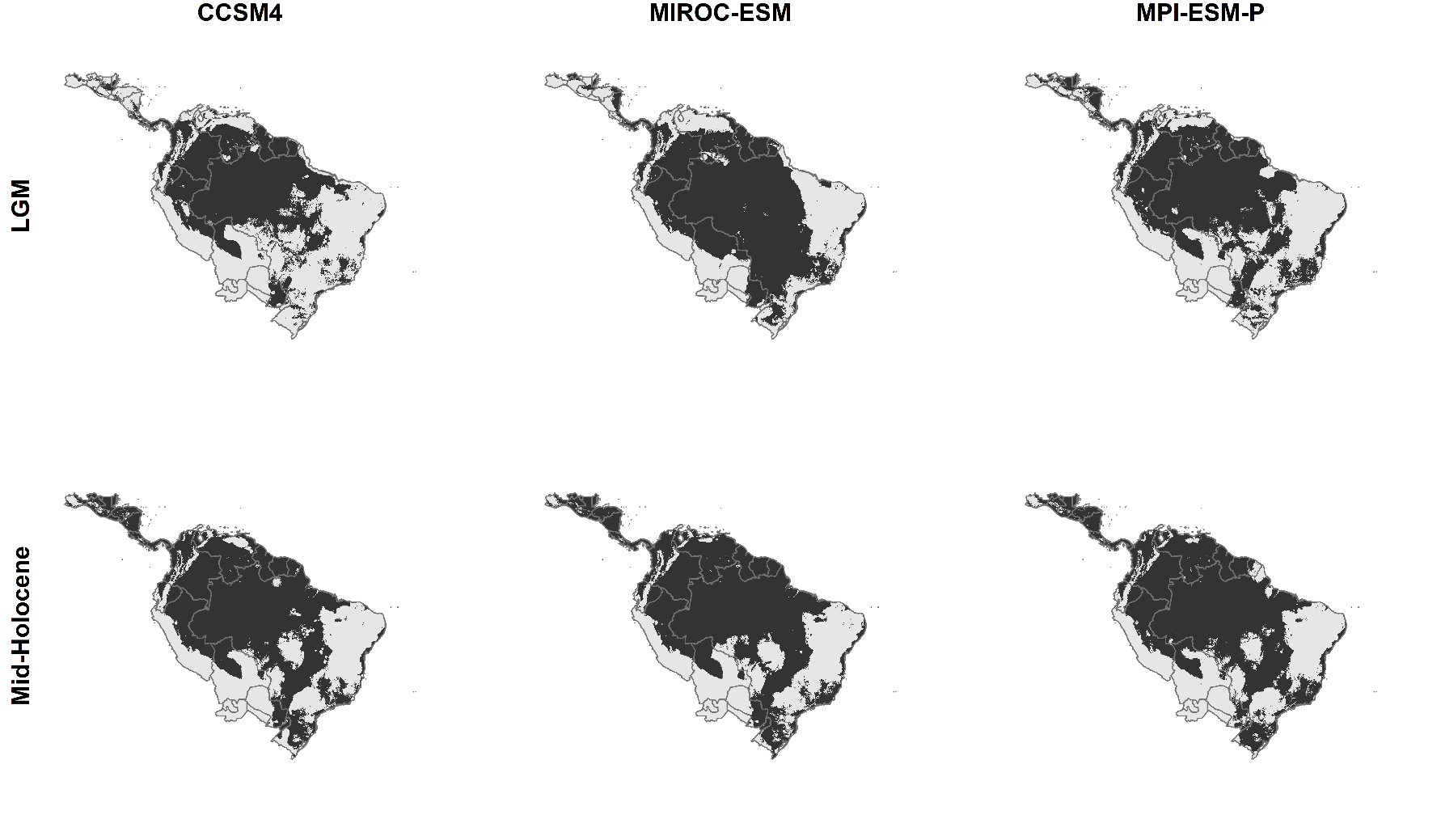
**

**Figure S6.** Predicted Paleo-distributions for the harpy eagle projected to the Last Glacial Maximum (LGM, ~22,000ya) and Mid-Holocene (~6,000ya) reclassified to binary predictions across three paleoclimate General Circulation Models (GCMs). Black areas are suitable environmental space above the 10TP threshold (0.415).


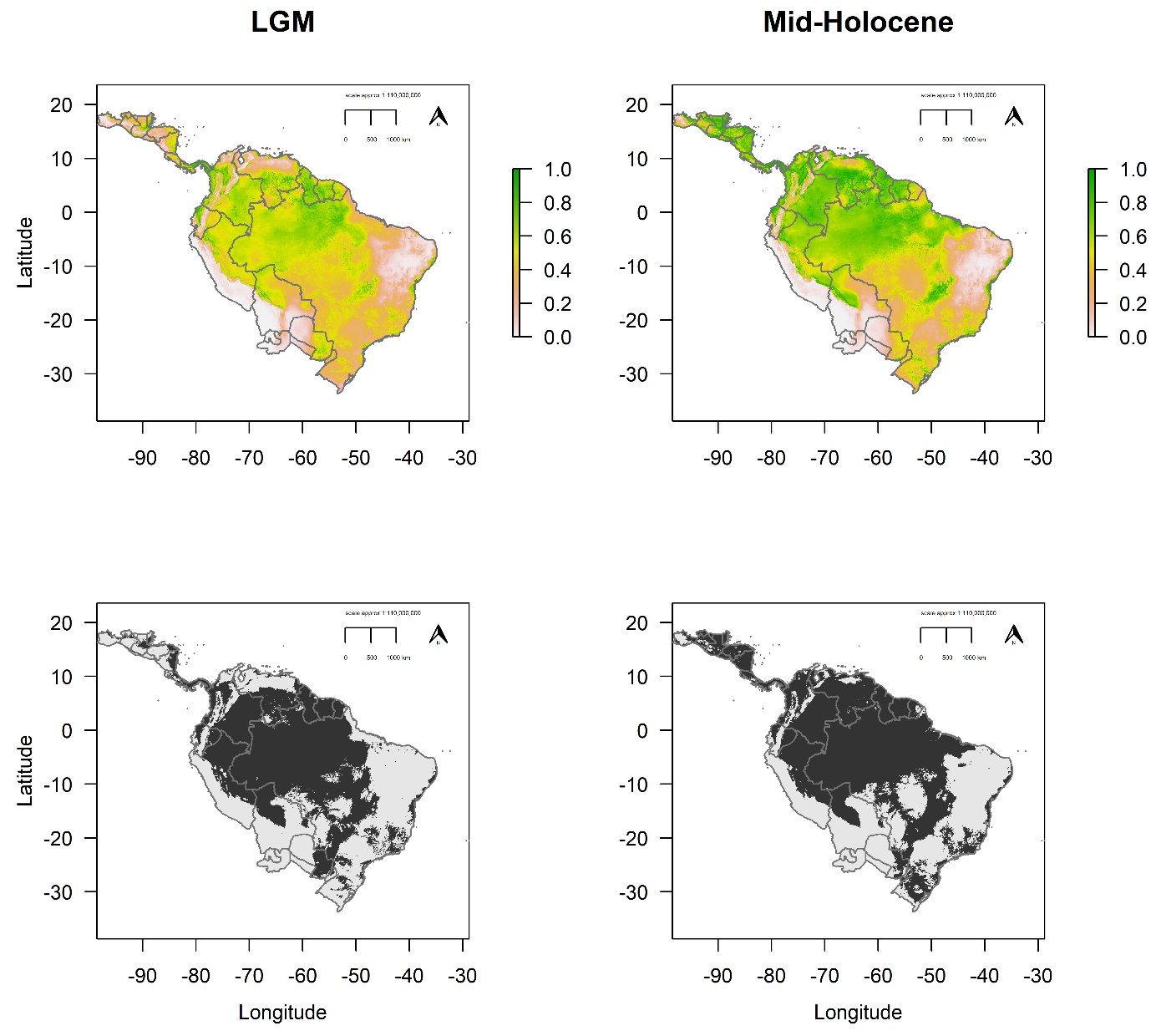


**Figure S7.** Predicted paleo-distributions for the harpy eagle projected to the Last Glacial Maximum (LGM, ~22,000 years ago) and Mid-Holocene (~6,000 years ago). Maps define the mean predictions from the three paleoclimate General Circulation Models masked to current geographic extent and geo-political boundaries.
